# Exploring the Relation of Harsh Parental Discipline with Child Emotional and Behavioral Problems by Using Multiple Informants. The Generation R Study

**DOI:** 10.1371/journal.pone.0104793

**Published:** 2014-08-13

**Authors:** Joreintje D. Mackenbach, Ank P. Ringoot, Jan van der Ende, Frank C. Verhulst, Vincent W. V. Jaddoe, Albert Hofman, Pauline W. Jansen, Henning W. Tiemeier

**Affiliations:** 1 The Generation R Study Group, Erasmus MC-University Medical Centre, Rotterdam, The Netherlands; 2 Department of Child and Adolescent Psychiatry/Psychology, Erasmus MC-University Medical Centre, Rotterdam, The Netherlands; 3 Department of Epidemiology, Erasmus MC-University Medical Centre, Rotterdam, The Netherlands; 4 Department of Pediatrics, Erasmus MC-University Medical Centre, Rotterdam, The Netherlands; 5 Department of Psychiatry, Erasmus MC-University Medical Centre, Rotterdam, The Netherlands; Hamamatsu University School of Medicine, Japan

## Abstract

Parental harsh disciplining, like corporal punishment, has consistently been associated with adverse mental health outcomes in children. It remains a challenge to accurately assess the consequences of harsh discipline, as researchers and clinicians generally rely on parent report of young children's problem behaviors. If parents rate their parenting styles and their child's behavior this may bias results. The use of child self-report on problem behaviors is not common but may provide extra information about the relation of harsh parental discipline and problem behavior. We examined the independent contribution of young children's self-report above parental report of emotional and behavioral problems in a study of maternal and paternal harsh discipline in a birth cohort. Maternal and paternal harsh discipline predicted both parent reported behavioral and parent reported emotional problems, but only child reported behavioral problems. Associations were not explained by pre-existing behavioral problems at age 3. Importantly, the association with child reported outcomes was independent from parent reported problem behavior. These results suggest that young children's self-reports of behavioral problems provide unique information on the effects of harsh parental discipline. Inclusion of child self-reports can therefore help estimate the effects of harsh parental discipline more accurately.

## Introduction

Parenting practices play a fundamental role in children's emotional and behavioral development. Corporal disciplining practices have consistently been associated with adverse mental health outcomes, such as poor school achievements, behavioral problems, lowered self-esteem and delinquent behaviors [1]–[4]. Milder forms of negative parental disciplining strategies -like harsh discipline- have also been studied repeatedly. Harsh discipline is characterized by parental attempts to control a child using verbal violence (shouting) or physical forms of punishment (pinching or hitting) [5]. These forms of parental disciplining practices have been associated not only with child behavioral problems, in line with a cycle of violence hypothesis [6], but also with child emotional problems [1], [5], [7], [8]. The effects of these milder forms of harsh disciplining may be less pronounced, yet are important since the prevalence of these forms of parental discipline is high. In a recent study using data from the present cohort we demonstrated that no less than 77% of mothers and 67% of fathers shouted at their child at least once in the last two weeks, in addition the number of parents threatening to slap (20–24%) or angrily pinching the child's arm (15%) was also considerable [9]. Given the high prevalence and the known burden for children it is important to examine the consequences of these milder forms of harsh parental disciplining accurately.

As child behavior problems like aggressive or oppositional behaviors may lead to higher levels of harsh discipline by parents [10], it is important to study the effects of harsh parental disciplining on child problem behaviors prospectively. A number of longitudinal studies have affirmed that, after controlling for baseline emotional and behavioral problems, children exposed to less extreme forms of parental harsh discipline have an increased risk of behavioral problems and psychiatric disorders later in life [2], [7].

Despite a large body of evidence, the existing literature on emotional and behavioral consequences of mild harsh discipline suffers limitations. Most studies relied on parental and often only on maternal report of child behavioral problems [2], [7], [11]–[13]. Relying on one informant for both the determinant and the outcome is problematic, as parents who rate their own parenting styles as ‘harsh’ may also perceive their child's behavior differently than parents that do not use harsh disciplining [5], [10], [14], [15]. This problem of shared informant bias can be avoided if the informant reporting on the consequences of harsh discipline differs from the informant reporting on harsh discipline. Including multiple reporters may generate additional evidence regarding the consequences of harsh discipline. It has become widely accepted that young children may be a valuable source of information [16], as they can provide unique insights into their own behaviors [17]. Indeed, self-report on the consequences of harsh discipline has proven to generate valuable results in adolescents [11], [18], [19]. Yet, few studies on the consequences of parental harsh disciplining have used young children's self-reports.

In the present study we examined the consequences of both maternal and paternal harsh discipline on parent reported and young children's self-reported emotional and behavioral problems. Specifically, we investigated whether any effect observed using child report was independent of parent reported problems. We hypothesized that child self-report of problem behavior would strengthen the evidence of an association between harsh discipline and parent reported problem behavior by contributing unique information.

## Methods

### Ethics statement

The study was conducted in accordance with the guidelines proposed in the World Medical Association Declaration of Helsinki and has been approved by the Medical Ethical Committee of the Erasmus Medical Center in Rotterdam, the Netherlands (MEC 198.782/2001/31). Full written informed consent for the postnatal phase was obtained from parents for both parental and child data.

### Study design and population

This study was embedded in the Generation R Study, a prospective population-based cohort from fetal life onwards. The design and data collection methods have been extensively described elsewhere [20]. Briefly, all pregnant women residing in Rotterdam, with an expected delivery date between April 2002 and January 2006, were eligible for participation in Generation R. For this study, we considered participants with full postnatal written consent (N = 7,295) eligible. A questionnaire including parental disciplining at age three years was returned by 4,733 mothers and constituted the baseline. Of those, 718 children had missing data on child self-reported emotional and behavioral problems (BPI) at age six years, yielding a sample size of 4,015 (follow-up response: 85%) for analyses with maternal harsh discipline and *child reported problems*. The sample size for analyses with maternal harsh discipline and *parent reported problems* was n = 3,764. A flow chart is provided in supplementary material (Figure S1).

### Measures

#### Harsh Discipline

Information about parental disciplining practices was obtained by postal questionnaires when the children were three years old. We assessed various types of disciplining by ten items that were based on the Parent-Child Conflict Tactics Scale [21]. In a previous study in the same cohort, a harsh discipline scale was confirmed using factor analysis. This resulted in a scale consisting of six items, representing constructs of psychological aggression and (mild) physical assault: “In the past week/month, I angrily pinched my child's arm”, “I shouted, yelled or screamed angrily at my child”, “I scolded at my child”, “I threatened to slap, spank or hit my child but did not actually do it”, “I called my child dumb or lazy or some other name like that” and “I shook my child”. Items were scored on a scale from 0 to 2. In line with this previous study [9], we calculated separate maternal and paternal harsh discipline scores by summing these six items. This yielded a score ranging from 0 to 12, with higher scores reflecting higher severity of harsh discipline.

#### Emotional and behavioral problems

Children were invited to our research centre in Rotterdam at the age of six years. During this visit, the Berkeley Puppet Interview (BPI) was used to assess emotional and behavioral problems as perceived by the child him/herself as described previously [22]. The BPI is a semi-structured interactive interview technique to obtain self-reports of young children. During the interview, two identical dog hand puppets were introduced to the child and invited the child to engage in a conversation. The puppets made opposing statements about themselves. For example, one puppet said that he was a sad kid, while the other puppet said the he was not a sad kid. Subsequently, the puppets asked children to indicate which statement described themselves best. In this study, we used internalizing (emotional problems) and externalizing (behavioral problems) scales. The Internalizing scale score (20 items) was computed as the sum of the item scores in three scales: Depression, Separation Anxiety and Overanxious. The Externalizing scale score (21 items) was computed as the sum of the item scores in three scales: Oppositional Defiant, Overt Hostility and Conduct Problems. Higher scores on the BPI scales indicate more problems. The psychometric properties of the BPI emotional and behavioral scales in the present study have been described elsewhere [22], [23].

Parent-reported child emotional and behavioral problems were assessed with the Dutch version of the Child Behavior Checklist (CBCL/1,5-5), a 99-item questionnaire that was mailed prior to the visit to the research centre [24]. One of the parents, usually the mother, completed the CBCL/1,5-5 just before the visit to the research centre (92% of the questionnaires were completed by the mothers, 8% by other (primary) caregivers). The internalizing (emotional problems) and externalizing (behavioral problems) broadband scales were used in the present study. The Internalizing scale score (36 items) is the sum of the item scores of four scales: Emotionally Reactive, Anxious/Depressed, Somatic Complaints, and Withdrawn. The Externalizing scale score (24 items) is the sum of the item scores of the Attention Problems and Aggressive Behavior scales. Higher scores on the CBCL scales indicated more problems. Good reliability and validity has been reported for the CBCL/1,5-5 [25].

Assessing child problems at age six years was considered appropriate, as both the BPI and the CBCL are valid tools to assess child emotions and behaviors at this age. [22], [26], [27] Pre-existing child internalizing and externalizing problems were reported by both mother and father using the CBCL/1,5-5 when children were 3 years old. This provided a three year difference, during transition from preschool to school-age, between determinant and outcome.

#### Covariates

Potential confounders were selected based on prior studies [7], [8], [28]. Information on gender, date of birth, marital status of the parents, smoking during pregnancy and age of the parents at intake was obtained from midwifery and hospital registries. Information on ethnicity, number of children in the household, educational level of the parents and household income was obtained by questionnaires at age 6 years. The child's ethnicity was classified by the countries of birth of the parents, according to the Dutch standard classification criteria of Statistics Netherlands (2004), and was categorized into Dutch, European and Non-western background (e.g. Turkish, Moroccan, Indonesian, Cape Verdian, Surinamese and Antillean). Educational level of the parents was defined as low (at most lower vocational training), medium low (at most intermediate vocational training), medium high (higher vocational training) and high (university degree). Family household income was divided into two categories: below 2,000 Euros per month which corresponds with below modal income, and 2,000 Euros per month and above. Marital status of parents was defined as either being married/living together or as having no partner.

To assess global parental psychopathology, a selection of 21 items from the Brief Symptom Inventory (BSI) [29] was administered to both mothers and fathers when the child was three years old.

Family functioning was measured with the 12-item General Functioning scale of the McMasters Family Assessment Device (FAD) [30]. In this validated self-report questionnaire, parents (in 82% of the cases this was the mother) rated family functioning and family stress on a 4-point scale.

### Statistical analyses

We first conducted descriptive analyses of the population. Next, correlational analysis of harsh parenting, emotional (internalizing) and behavioral (externalizing) problems and parental psychopathology was performed.

The relation between harsh discipline at age three years and emotional and behavioral problems at age six years was examined with linear regression analyses. To satisfy the assumption of normality, maternal and paternal harsh discipline scores were square root transformed to achieve a normal distribution. Similarly, BPI and CBCL scale scores were transformed using the natural logarithm and the square root respectively, and z-scores were calculated to be able to compare the emotional and behavioral problems with each other.

We studied the effects of maternal and paternal harsh discipline separately. Similarly, we studied parent and child self-reports of emotional and behavioral problems as separate outcomes.

In model 1, unadjusted linear regression analyses of harsh discipline with child emotional and behavioral problems were performed. In model 2, we adjusted for sociodemographic characteristics (child gender, age and ethnicity, number of children in the household, household income, marital status, smoking during pregnancy, maternal and paternal educational level), maternal and paternal psychopathology score, and family functioning. Covariates were included in the second model if they changed the effect estimates of the unadjusted relation between harsh discipline and emotional and behavioral problems by more than 5%. (However, had we used a 10% change in effect estimates as inclusion criterion -another commonly used criterion [31]- the same confounders would have been selected.) To adjust for pre-existing emotional and behavioral problems, in model 3 we additionally accounted for emotional (if emotional problems were the outcome) or behavioral problems (if behavioral problems were the outcome) assessed at age 3 years. If the association between harsh discipline and problem behavior is independent of baseline problem behavior, this would strengthen the assumption that the temporality of the associations.

Analyses were adjusted for maternal characteristics (maternal education, maternal psychopathology and the maternal report of pre-existing child emotional/externalizing problems) unless paternal harsh discipline was the independent variable; in this case we adjusted for the respective paternal characteristics.

Next, we additionally adjusted the analyses of the child self-report problem behavior (model 3) for parent reported emotional and behavioral problems (model 4). The aim of this analysis was to examine whether harsh discipline could predict child self-reported emotional and behavioral problems, over and above parent report. If the association is independent of parent report, this suggests children can provide unique outcome information in this study of parental harsh discipline.

To test the influence of effect modifiers, we specified interaction terms for harsh discipline with child gender, child ethnicity and a mutual interaction term between maternal harsh discipline and paternal harsh discipline on the risk of emotional and behavioral problems. None of the interaction terms was statistically significant.

Missing values on the covariates were estimated using multiple imputation techniques and were based on available information on determinants, outcome and covariates of this study. The presented results are based on pooled estimates of ten imputed datasets [32].

Analyses were conducted in the number of children with data available for the outcome of interest (for example parent reported emotional problems). As we did not impute the outcome variables the number of children per analysis differed from 3,047 to 4,015. We repeated all analyses in participants with complete data (N = 3,047). Linear regression analyses were performed using the SPSS version 18.0 (SPSS Inc., Chicago, IL).

### Baseline nonresponse and loss-to-follow up analysis

In total, 4,733 mothers completed the questionnaire on harsh discipline at baseline. Mothers (N = 2,562) who did not complete this questionnaire were on average younger (28.6 years versus 31.5 years, *F(2, N = 7,295)* = 11.8, *p*<.001), and were more likely to have continued smoking during pregnancy (20.8% versus 12.0%, *χ^2^*(2, *N* = 7,295) = 501.0, *p*<.001), to have a family income below modal (32.7% versus 15.4%, *χ^2^*(1, *N* = 7,295) = 238.5 *p*<.001) and to have no partner (21.4% versus 8.2%, *χ^2^*(1, *N* = 7295) = 231.8, *p*<.001) than mothers who completed the questionnaire.

At follow-up, when the child was between five and eight years old, 4,015 children (85%) of the 4,733 mothers who returned the questionnaire at age three, completed the Berkeley Puppet Interview. We compared these families with families of children who did not complete the BPI (N = 718). Children without a BPI assessment were more likely to be of non-Dutch origin (38.3% versus 32.2%, *χ^2^*(2, *N* = 4733) = 11.8, *p* = .003), but did not differ from their peers who completed a BPI assessment in terms of maternal harsh discipline score (2.2 versus 2.2, *F* = 0.7, *p* = 0.63), behavioral problems at age three (5.4 versus 5.0, *F* = 10.2, *p* = .08), parent reported behavioral problems at age six (7.5 versus 6.9, *F* = 12.4, *p* = .13) or family income (14.3% versus 15.6%, below modal, *χ^2^*(1, *N* = 4733)* = *0.8, *p* = .40).

## Results

Characteristics of the study sample are presented in Table 1. Children had a mean age of 3.1 years at baseline and a mean age of 6.1 years at follow-up. Sixty-seven percent of the children were of Dutch origin, 8.2% had a European and 24.4% a Non-western background.

**Table 1:** Characteristics of the study population.

Child & family characteristics	N = 4,015	Percentages or means (sd)
Gender (% boys)		*49.9%*
Age at BPI^¥^ measurement in years		6.03 (0.35)
Child ethnicity		
Dutch		*67.4%*
European		*8.2%*
Non-Western		*24.4%*
Number of children in the household		2.50 (1.72)
Age mother at intake		31.69 (4.54)
Age partner at intake		34.09 (5.28)
Harsh discipline by mother		2.18 (1.95)
Harsh discipline by father		1.82 (1.81)
Household income (% above modal)		*68.7%*
Marital status (% with partner)		*92.8%*
Highest educational level of parents		
Low		*4.4 %*
Medium low		*19.9 %*
Medium high		*26.5 %*
High		*49.3 %*
Smoking during pregnancy		
Never		*79.3 %*
Until pregnancy was known		*9.1 %*
Continued during pregnancy		*11.6 %*
Family functioning score		1.49 (0.41)
Psychopathology of mother score		3.43 (5.62)
Psychopathology of father score		2.71 (4.45)
Parent reported CBCL scores		
Emotional problems score† at age 3		5.15 (4.39)
Behavioral problems score† at age 3		8.76 (5.71)
Emotional problems score† at age 6		5.44 (5.28)
Behavioral problems score† at age 6		6.94 (5.28)
Child self-reported BPI scores		
Emotional problems score‡ at age 6		58.16 (12.10)
Behavioral problems score‡ at age 6		51.92 (10.60)

† measured by the Child Behavior Checklist (parent report).

‡ measured by the Berkeley Puppet Interview (child self-report).

Table 2 shows the Pearson correlation coefficients between harsh discipline, the different emotional and behavioral problem scales, and parental psychopathology. Parent reports of emotional and behavioral problems were highly correlated (*r* at age three = 0.61, *p*<.001, *r* at age six = 0.66, *p*<.001), whereas child reported emotional and behavioral problems were less strongly correlated (*r* = 0.30, *p*<.001). Parent and child reports of behavioral problems were more strongly correlated (*r* = 0.18, *p*<.001) than emotional problems (*r* = 0.10, *p*<.001). Maternal and paternal harsh discipline was correlated to all emotional and behavioral scales, with the exception that there was no correlation between paternal harsh discipline and child reported emotional problems.

**Table 2:** Correlational analysis of harsh discipline, emotional and behavioral problems and parental psychopathology.

	Mean	*SD*	1.	2.	3.	4.	5.	6.	7.	8.	9.	10.
1. CBCL† emotional sumscore at age 3‡	5.09	3.86	1									
2. CBCL† behavioral sumscore at age 3‡	8.79	5.27	0.61**	1								
3. CBCL† emotional sumscore at age 6	5.41	5.24	0.49**	0.40**	1							
4. CBCL† behavioral sumscore at age 6	6.92	6.05	0.35**	0.54**	0.66**	1						
5. BPI¥ emotional sumscore at age 6	57.95	12.08	0.08**	0.07**	0.10**	0.08**	1					
6. BPI¥ behavioral sumscore at age 6	51.84	10.56	0.03*	0.13**	0.07**	0.18**	0.30**	1				
7. Maternal harsh discipline	2.16	1.91	0.22**	0.35**	0.19**	0.26**	0.05*	0.09*	1			
8. Paternal harsh discipline	1.80	1.81	0.20**	0.37**	0.12**	0.23**	0.02	0.11*	0.38*	1		
9. Maternal psychopathology	3.33	5.39	0.32**	0.30**	0.28**	0.26**	0.05*	0.04*	0.30*	0.12*	1	
10. Paternal psychopathology	2.68	4.39	0.30**	0.28**	0.16**	0.17**	0.06*	0.06*	0.12*	0.26*	0.28*	1

† Child Behavior Checklist (parent report).

‡ Mean of mother and father score on the internalizing/externalizing scale.

¥ Berkeley Puppet Interview (child self-report).

** p-value <0.001.

* p-value <0.05.

Table 3 shows the results of the linear regression analyses with *behavioral problems* as outcome. First, we assessed the relation between harsh discipline and *parent reported behavioral problems* (CBCL). Adjustment for sociodemographic covariates and family characteristics attenuated the effect of harsh discipline on behavioral problems. Additional adjustment for baseline behavioral problems at age three further attenuated the effect estimates, but the relation between *maternal* harsh discipline and parent reported behavioral problems remained (model 3: *B* =  0.06, *95%CI*: 0.02, 0.09). Analyses of the relation between *paternal* harsh discipline and behavioral problems yielded similar results (model 3: *B* =  0.08, *95%CI*: 0.04, 0.13).

**Table 3:** Effect of harsh discipline on behavioral problems.

	Behavioral problems								
	Parent report‡					Child report†			
	N		B	95%CI	R^2^	N	B	95%CI	R^2^
Harsh discipline (HD) by the mother									
Model 1 - HD score mother	3773		**0.34**	0.30, 0.38		3998	**0.12**	0.08, 0.16	
Model 2$ - HD score mother, adjusted model			**0.24**	0.20, 0.29			**0.10**	0.06, 0.14	
Model 3□ - HD score mother fully adjusted model			**0.05**	0.02, 0.09	0.36		**0.07**	0.03, 0.11	0.05
Harsh discipline (HD) by the father									
Model 1 - HD score father	3051		**0.29**	0.24, 0.33		3172	**0.14**	0.10, 0.18	
Model 2$ - HD score father, adjusted model			**0.21**	0.17, 0.26			**0.11**	0.07, 0.15	
Model 3□ - HD score father fully adjusted model			**0.08**	0.04, 0.13	0.25		**0.09**	0.04, 0.13	0.05

**Bold** numbers represent statistically significant (p<0.05) associations.

‡ Z-score of the Child Behavior Checklist/1,5-5 measured at age 6.

† Z-score of the Berkeley Puppet Interview measured at age 6.

$ Model adjusted for ethnicity of the child, gender of the child, age of the child, number of children in the household, household income, marital status, highest education of the parents, smoking during pregnancy, parental psychopathology and parental report of family functioning.

□ Model additionally adjusted for baseline internalizing/externalizing behavior at age three.

Analyses with *child self-reported behavioral problems* (BPI) showed that higher levels of maternal harsh discipline were associated with higher levels of child reported behavioral problems. Although effect sizes were somewhat smaller than those for parent reported problems, the overall pattern for child reported behavioral problems across the three models was very similar to the effect observed if based on parent report. Even after adjustment for all covariates, *maternal* and *paternal* harsh discipline were associated with a higher score on child self-reported behavioral problems (model 3: *B* for maternal harsh discipline = 0.07, *95%CI*: 0.03, 0.11; *B* for paternal harsh discipline = 0.07, *95%CI*: 0.03, 0.12).

Table 4 shows the relation between harsh discipline and *emotional problems*. Higher levels of maternal and paternal harsh discipline were associated with more parent reported emotional problems (model 3: *B* for maternal harsh discipline = 0.06, *95%CI*: 0.03, 0.10, model 3; *B* for paternal harsh discipline = 0.04, *95%CI*: 0.00, 0.08). Yet, we found that neither maternal nor paternal harsh discipline was related to emotional problems as reported by the child in model 3 (*B* for maternal harsh discipline = 0.02, *95%CI*: -0.02, 0.06; *B* for paternal harsh discipline = 0.01, *95%CI*: -0.03, 0.006).

**Table 4:** Effect of harsh discipline on emotional problems.

			Emotional problems						
		Parent report‡				Child report†			
		N	B	95%CI	R^2^	N	B	95% CI	R^2^
Harsh discipline (HD) by the mother									
Model 1 - HD score mother		3764	**0.24**	0.20, 0.28		4015	**0.07**	0.03, 0.11	
Model 2$ - HD score mother, adjusted model			**0.13**	0.09, 0.17			0.03	-0.01, 0.07	
Model 3□ - HD score mother fully adjusted model			**0.05**	0.01, 0.08	0.31		0.03	-0.02, 0.07	0.06
Harsh discipline (HD) by the father									
Model 1 - HD score father		3047	**0.15**	0.10, 0.19		3182	0.03	-0.01, 0.07	
Model 2$ - HD score father, adjusted model			**0.08**	0.04, 0.13			0.03	-0.02, 0.07	
Model 3□ - HD score father fully adjusted model			**0.04**	0.00, 0.08	0.19		0.02	-0.02, 0.07	0.04

**Bold** numbers represent statistically significant (p<0.05) associations.

‡ Z-score of the Child Behavior Checklist/1,5-5 measured at age 6.

† Z-score of the Berkeley Puppet Interview measured at age 6.

$ Model adjusted for ethnicity of the child, gender of the child, age of the child, number of children in the household, household income, marital status, highest education of the parents, smoking during pregnancy, parental psychopathology and parental report of family functioning^.^

□ Model additionally adjusted for baseline internalizing/externalizing behavior at age three.

To test whether the association of harsh discipline with child self-reported behavioral problems was independent of parent report, we additionally adjusted this relation for *parent* reports of behavioral problems. Both maternal and paternal harsh discipline predicted child reported behavioral problems, independently of parent reported behavioral problems (*B* for maternal harsh discipline = 0.06, *95%CI*: 0.02, 0.10, R^2^ = 0.06; *B* for paternal harsh discipline = 0.06, *95%CI*: 0.02, 0.11, R^2^ = 0.06).

The above analyses were conducted in the number of children with data available for one or more of the outcome measures to reduce selection bias. Next, we repeated all analyses in those participants with complete data to allow for optimal comparison between analyses. Results were essentially unchanged.

## Discussion

Parental harsh discipline -whether used by father or mother- increases the risk of behavioral problems in young children. In the present study, mild forms of harsh parental discipline were negatively associated with parent and child reported behavioral problems. By adjusting for pre-existing problems, we showed that this reflects an increase in problems across a three-year period. Most importantly, we demonstrated that children provide independent information when assessing the effects of parental harsh discipline on behavioral problems, whereas the results for child and parent-reported emotional problems were less consistent.

Studies have repeatedly associated harsh disciplining practices based on parent reports of child emotional and behavioral problems (i.e. [2], [7]). However, in the present study the effects of harsh discipline on behavioral problems were not restricted to harsh discipline by the father, as proposed by Avakame [6] and reported by Chang et al. [5]. Rather, maternal harsh discipline had effects very comparable to harsh discipline of the father. Possibly, the disciplining tactics we studied were mild and verbally oriented (e.g. screaming and threatening) and may thus not discriminate well between maternal and paternal disciplining tactics. Clear differences between mothers and fathers may be detected only if more extreme forms of harsh disciplining are studied. Alternatively, the presence of any harsh behavior in a family is more important than the gender of the disciplining parent. Indeed, partners are often similar in antisocial behavior [33], i.e., mothers who tend to discipline their children harshly more often have partners who also practice this parenting discipline.

Our findings based on child self-reported behavioral problems were not only consistent with those from parent reported behavioral problems, but effects observed using child reports were independent of the parental report. Increased explained variance underpinned this finding. This supports our hypothesis and suggests that children provide unique information on the behavioral consequences of harsh parenting. This observation is clinically relevant since parents using harsh disciplining strategies may interpret their children's behavior differently than other parents [5], [10], [14], [15]. These biased reports may come about for a number of reasons. For example, parents may report higher levels of child problems in their own defense due to emotional overinvolvement. Alternatively, highly critical parenting may cause some parents to have a low tolerance for otherwise normal child problems due to stress. Lastly, from the perspective of authoritarian parenting, certain parents tend to notice only the most extreme behaviors. [15]

Concluding, if all information (on outcome and determinant) is obtained from one informant, reporter bias may occur [15]. The results from this study indicate young children may be considered as one of the sources of information in a multi-informant approach on the consequences of harsh parenting. Child self-reports not only confirmed the parental reports but suggest that scientists may underestimate the effect of harsh parenting as the child provided independent information on the possible behavioral consequences of harsh parenting.

Maternal *and* paternal harsh discipline were associated with emotional problems as reported by the primary caregiver but not with emotional problems reported by the child. This was in contrast to rather similar effect sizes observed for the association of harsh parenting with child and parent reported behavioral problems. A relation between harsh discipline and emotional problems in children was hypothesized as children can develop a negative view of the self and feel worthless as a result of a harsh parental discipline style [34]. On the other hand, emotional problems are determined more by genetic variations and less by role modeling than behavioral problems [35], [36]. However, some methodological explanations for the discrepancy in the analyses using between parent and child reports of emotional problems must be discussed. First, parents distinguish less between emotional and behavioral problems than children. The correlations between parent reported emotional and behavioral problems are higher than those of child reported emotional and behavioral problems (.66 vs .30 in this study). The effect of harsh discipline on parent reported emotional problems may partly reflect the association between harsh discipline and behavioral problems. Second, children may be less accurate reporters of emotional problems [23] as these are less concrete aspects of behavior [37]. Third, children's ideas about the self do not necessarily match with objectively observable constructs. The concordance between self-perceptions and observable behavior may grow stronger when children age [38]. Therefore, we recommended studying the children's perspective on the consequences of harsh discipline over a longer period of time. Finally, these inconsistent results support findings from previous studies suggesting that all informants' reports are imperfect measures of child behavior [39]. Therefore, combining information from multiple sources is considered most optimal [40].

### Strengths and limitations

Some methodological considerations need to be taken into account. Strengths of this study are the large number of population-based participants. In addition, we obtained both parent and child reports of emotional and behavioral problems at age six, and reports of both maternal and paternal harsh discipline were available. Adjusting for baseline problems (pre-existing child problems at age three) allowed us to analyse changes in emotional and behavioral problems in a relatively short period of time.

One of the limitations of our study is that, although we included large numbers of participants, non-response analysis showed some selective attrition. This resulted in an under-representation of children from families with a lower income and mothers without a partner, while families from a low socioeconomic background are at increased risk for both parental harsh discipline [9] and child behavioral problems [41]. However, although prevalence rates have an impact on statistical power, these changes do not necessarily alter the relationship between determinant and outcome [42].

A second limitation is that we had to rely on parent reports only of baseline child problems. Adjusting child self-reported problems for baseline parent reported child problems is not the optimal adjustment to rule out reverse causality. However, it is not feasible to conduct interviews in three-year old children about their behavior because the BPI and other child self-report instruments only yield reliable estimates in children from older ages [43]. Although the primary caregiver was asked to fill out the questionnaire assessing child behavior at age six, mostly mothers completed the questionnaire. Therefore, parent reported child emotional and behavioral problems mostly reflected the mothers' views of child problem behavior.

“Third, when parents report on their own harsh disciplining, social desirability may lead to a response bias. Even though, in this study only mild forms of harsh discipline were investigated - as three items on the physical assault scale were excluded from the questionnaire [44] - parental underreporting of any verbal or psychological tactics may have been the case. Yet, misclassification in the group of parents that did report harsh disciplining is less likely: if parents reported harsh disciplining tactics, this has most probably been the case. Taken together, these response patterns may have resulted in an underestimation of the effect.”

Fourth, emotional and behavioral problems were assessed differently between children and parents as items in the CBCL-questionnaire differ from items in the BPI interview. However, both measures are accepted ways of assessing child emotional and behavioral problems. [22], [25]

### Implications

Our study confirmed that even mild forms of harsh parental discipline have substantial effects on the behavioral development of a child. Importantly, this study showed that young children can provide independent, valuable information on behavioral problems as a result of harsh disciplining styles. Although information from young children should be treated with some caution, the possibility to obtain information from very young children provides opportunities for instances when parents are unavailable or unwilling to serve as informants on emotional or behavioral consequences of their parenting behavior. In general, child self-report could be used in addition to caregiver report when assessing problem behavior, because both the perspective of the child and the parent is important.

The current findings have implications for programs that aim to identify and provide support for children at risk of, or experiencing, harsh discipline. Health care workers should be well aware of the effects of even mild harsh discipline on behavioral problems in children.

## Supporting Information

Figure S1 **Flowchart of study participants.**
(DOCX)

## References

[1] Teicher MH, Samson JA, Polcari A, McGreenery CE (2006) Sticks, stones, and hurtful words: relative effects of various forms of childhood maltreatment. Am J Psychiatry 163: 993–1000. 16741199 10.1176/ajp.2006.163.6.993

[2] Larzelere RE (2000) Child outcomes of nonabusive and customary physical punishment by parents: an updated literature review. Clin Child Fam Psychol 3: 199–221. 10.1023/a:1026473020315 11225737

[3] Sidebotham P, Golding J (2001) Child maltreatment in the “children of the nineties” a longitudinal study of parental risk factors. Child Abus Negl 25: 1177–1200. 10.1016/s0145-2134(01)00261-7 11700691

[4] Berger LM (2005) Income, family characteristics, and physical violence toward children. Child Abus Negl 29: 107–133. 10.1016/j.chiabu.2004.02.006 15734178

[5] Chang L, Schwartz D, Dodge KA, McBride-Chang C (2003) Harsh parenting in relation to child emotion regulation and aggression. J Fam Psychol 17: 598–606. 14640808 10.1037/0893-3200.17.4.598 PMC2754179

[6] Avakame EF (1998) Intergenerational transmission of violence, self-control, and conjugal violence: a comparative analysis of physical violence and psychological aggression. Violence Vict 13: 301–316. 9836416

[7] Taylor CA, Manganello JA, Lee SJ, Rice JC (2010) Mothers' spanking of 3-year-old children and subsequent risk of children's aggressive behavior. Pediatrics 125: e1057–65. 20385647 10.1542/peds.2009-2678 PMC5094178

[8] Vostanis P, Graves A, Meltzer H, Goodman R, Jenkins R, et al (2006) Relationship between parental psychopathology, parenting strategies and child mental health—findings from the GB national study. Soc Psychiatry Psychiatr Epidemiol 41: 509–14. 16572271 10.1007/s00127-006-0061-3

[9] Jansen PW, Raat H, Mackenbach JP, Hofman A, Jaddoe VWV, et al (2012) Early Determinants of Maternal and Paternal Harsh Discipline: The Generation R Study. Fam Relat 61: 253–270.

[10] Anderson KE, Lytton H, Romney DM (1986) Mothers' interactions with normal and conduct-disordered boys: Who affects Whom? Dev Psychol 22: 604–609.

[11] Lansford JE, Dodge KA, Malone PS, Oburu P, Palme K, et al (2005) Physical Discipline and Children's Adjustment: Cultural Normativeness as a Moderator. Child Dev 76: 1234–1246. 16274437 10.1111/j.1467-8624.2005.00847.x PMC2766084

[12] Prinzie P, Onghena P, Hellinckx W (2006) A cohort-sequential multivariate latent growth curve analysis of normative CBCL aggressive and delinquent problem behavior: Associations with harsh discipline and gender. Int J Behav Dev 30: 444–459.

[13] McLeod JD, Kruttschnitt C, Dornfeld M (1994) Does parenting explain the effects of structural conditions on children's antisocial behavior? A comparison of Blacks and Whites. Soc Forces 73: 575–604.

[14] Fox RA, Platz DL, Bentley KS (1994) Maternal factors related to parenting practices, developmental expectations, and perceptions of child behavior problems. J Genet Psychol Res Theory Hum Dev 156: 431–441. 10.1080/00221325.1995.9914835 8543930

[15] Reid JB, Kavanagh K, Baldwin DV (1987) Abusive parents' perceptions of child problem behaviors: An example of parental bias. J Abnorm Child Psychol 15: 457–466. 3668090 10.1007/BF00916461

[16] Kraemer HC, Measelle JR, Ablow JC, Essex MJ, Thomas Boyce, et al (2003) A new approach to integrating data from multiple informants in psychiatric assessment and research: mixing and matching contexts and perspectives. Am J Psychiatry 160: 1566–1577. 12944328 10.1176/appi.ajp.160.9.1566

[17] Arsenault L, Moffitt TE, Caspi A, Taylor A, Rijsdijk FV, et al (2003) Strong genetic effects on cross-situational antisocial behaviour among 5-year-old children according to mothers, teachers, examiner-observers, and twins' self-reports. J Child Psychol Psychiatry 44: 832–848. 12959492 10.1111/1469-7610.00168

[18] Bolger KE, Patterson CJ (2001) Pathways from child maltreatment to internalizing problems: perceptions of control as mediators and moderators. Dev Psychopathol 13: 913–40. 11771914

[19] Lansford JE, Deater-Deckard K, Dodge KA, Bates JE, Pettit GS (2004) Ethnic differences in the link between physical discipline and later adolescent externalizing behaviors. J Child Psychol Psychiatry 45: 801–812. 15056311 10.1111/j.1469-7610.2004.00273.x PMC2772061

[20] Jaddoe VW, van Duijn CM, Franco OH, van der Heijden AJ, van IJzendoorn MH, et al (2012) The Generation R Study: design and cohort update 2012. Eur J Epidemiol 27: 739–56. 23086283 10.1007/s10654-012-9735-1

[21] Straus MA, Hamby SL, Moore DW, Runyan D (1998) Identification of child maltreatment with the Parent-Child Conflict Tactics Scales: development and psychometric data for a national sample of American parents. Child Abus Negl 22: 249–70. 10.1016/s0145-2134(97)00174-9 9589178

[22] Ringoot AP, Jansen PW, Steenweg-de Graaff J, Measelle JR, van der Ende J, et al (2013) Young children's self-reported emotional, behavioral, and peer problems: The Berkeley Puppet Interview. Psychol Assess 25: 1273–1285. 23937536 10.1037/a0033976

[23] Luby JL, Belden A, Sullivan J, Spitznagel E (2007) Preschoolers' contribution to their diagnosis of depression and anxiety: Uses and limitations of young child self-report of symptoms. Child Psychiatry Hum Dev 38: 321–338. 17620007 10.1007/s10578-007-0063-8

[24] Achenbach TM, Rescorla LA (2001) Manual for the ASEBA Preschool Forms & Profiles. Burlington: University of Vermont, Research Center for Children, Youth & Families.

[25] Tick NT, Ende J, van der, Koot HM, Verhulst FC (2007) 14-Year changes in emotional and behavioral problems of Dutch very young children. J Am Acad Child Adolesc Psychiatry 46: 1333–1340. 17885575 10.1097/chi.0b013e3181337532

[26] Rescorla L, Achenbach T, Ivanova MY, Dumenci L, Almqvist F, et al (2007) Behavioral and Emotional Problems Reported by Parents of Children Ages 6 to 16 in 31 Societies. J Emot Behav Disord 15: 130–142.

[27] Verlinden M, Veenstra R, Ringoot AP, Jansen PW, Raat H, et al (2014) Detecting bullying in early elementary school with a computerized peer-nomination instrument. Psychol Assess 26: 628–41. 24512423 10.1037/a0035571

[28] Day RD, Peterson GW, Mccracken C (2011) Predicting Spanking of Younger and Older Children by Mothers and Fathers. J Marriage Fam 60: 79–94.

[29] Boulet J, Boss MW (1991) Reliability and validity of the Brief Symptom Inventory. Psychol Assess 3: 433–437.

[30] Miller IW, Epstein NB, Bishop DS, Keitner GI (1985) The McMaster Family Assessment Device: reliability and validity*. J Marital Fam Ther 11: 345–356.

[31] Walter S, Tiemeier H (2009) Variable selection: current practice in epidemiological studies. Eur J Epidemiol 24: 733–736. 19967429 10.1007/s10654-009-9411-2 PMC2791468

[32] Schafer JL (1999) Multiple imputation: a primer. Stat Methods Med Res 8: 3–15. 10347857 10.1177/096228029900800102

[33] Zwirs B, Verhulst F, Jaddoe V, Hofman A, Mackenbach J, et al (2011) Partner similarity for self-reported antisocial behaviour among married, cohabiting and dating couples: the Generation R Study. Psychol Crime Law 18: 335–349.

[34] Sachs-Ericsson N, Verona E, Joiner T, Preacher KJ (2006) Parental verbal abuse and the mediating role of self-criticism in adult internalizing disorders. J Affect Disord 93: 71–8. 16546265 10.1016/j.jad.2006.02.014

[35] van der Valk JC, van den Oord EJCG, Verhulst FC, Boomsma DI (2003) Genetic and environmental contributions to stability and change in children's internalizing and externalizing problems. J Am Acad Child Adolesc Psychiatry 42: 1212–1220. 14560171 10.1097/00004583-200310000-00012

[36] van den Oord EJCG, Verhulst FC, Boomsma DL (1996) A genetic study of maternal and paternal ratings of problem behaviors in 3-year-old twins. J Abnorm Psychol 105: 349–357. 8772005 10.1037//0021-843x.105.3.349

[37] Harter S (1990) Adolescent self and identity development. In: Feldman SS, Ellio GR, At the treshold: the developing adolescent. Cambridge, MA: Harvard University Press. pp. 352–387.

[38] Davis-Kean PE, Huesmann LR, Jager J, Collins WA, Bates JE, et al (2008) Changes in the relation of self-efficacy beliefs and behaviors across development. Child Dev 79: 1257–1269. 18826524 10.1111/j.1467-8624.2008.01187.x

[39] Arseneault L, Kim-Cohen J, Taylor A, Caspi A, Moffitt TE (2005) Psychometric Evaluation of 5- and 7-Year-Old Children's Self-Reports of Conduct Problems. J Abnorm Child Psychol 33: 537–50. 16195949 10.1007/s10802-005-6736-5

[40] Kraemer HC, Measelle JR, Ablow JC, Essex MJ, Boyce WT, et al (2003) A new approach to integrating data from multiple informants in psychiatric assessment and research: mixing and matching contexts and perspectives. Am J Psychiatry 160: 1566–1577. 12944328 10.1176/appi.ajp.160.9.1566

[41] Bradley RH, Corwyn RF (2002) Socioeconomic status and child development. Annu Rev Psychol 53: 371–99. 11752490 10.1146/annurev.psych.53.100901.135233

[42] Wolke D, Waylen A, Samara M, Steer C, Goodman R, et al (2009) Selective drop-out in longitudinal studies and non-biased prediction of behaviour disorders. Br J Psychiatry 195: 249–56. 19721116 10.1192/bjp.bp.108.053751 PMC2802508

[43] Measelle JR, Ablow JC, Cowan PA, Cowan CP (1998) Assessing Young Children's Views of Their Academic, Social, and Emotional Lives: An Evaluation of the Self-Perception Scales of the Berkeley Puppet Interview. Child Dev 69: 1556–1576. 9914640

[44] Jansen PW, Raat H, Mackenbach JP, Hofman A, Jaddoe VWV, et al (2012) Early Determinants of Maternal and Paternal Harsh Discipline: The Generation R Study. Fam Relat 61: 253–270.

