# Supplementary material for: Exploring the Relation of Harsh Parental Discipline with Child Emotional and Behavioral Problems by Using Multiple Informants. The Generation R Study
Source: PLoS One. 2014 Aug 13;9(8):e104793. doi: 10.1371/journal.pone.0104793 (PMC4132073; doi:10.1371/journal.pone.0104793)
Supplement: Figure S1 — Flowchart of study participants. (DOCX) [file pone.0104793.s001.docx]

**Supplementary figure 1. Flowchart of study participants**

Numbers in the method section give the N for emotional problems

**Eligible population**

7,295 eligible participants with postnatal consent

*no information on maternal harsh discipline (N=2,551)*

**Baseline**

4,733 participants with information on *maternal* harsh discipline (baseline response 65%)

(of which 3,669 with information on *paternal* harsh discipline)

*Loss to follow-up*

*no assessment of the Berkeley Puppet Interview (N=729)*

**Follow-up**

Participants with Berkeley Puppet Interview data

*Child report of emotional problems* N=4,015

(follow-up response: 85%)

*Child report of behavioral problems* N=3,998

(of which N=3,182 and N=3,172 with information on *paternal harsh discipline*)

Participants with both information on the Berkeley Puppet Interview and the Child Behavior Checklist

*Parent report of emotional problems* N=3,764

*Parent report of behavioral problems* N=3,773

(of which N=3,047 and N=3,051 with information on *paternal harsh discipline*)

*no assessment of the Child Behavior Checklist (N=242)*
